# Identification of Differentially Expressed Genes Associated with Prognosis of B Acute Lymphoblastic Leukemia

**DOI:** 10.1155/2015/828145

**Published:** 2015-02-24

**Authors:** Idalia Garza-Veloz, Margarita L. Martinez-Fierro, Jose Carlos Jaime-Perez, Karol Carrillo-Sanchez, Maria Guadalupe Ramos-Del Hoyo, Angel Lugo-Trampe, Augusto Rojas-Martinez, Cesar Homero Gutierrez-Aguirre, Oscar Gonzalez-Llano, Rosario Salazar-Riojas, Alfredo Hidalgo-Miranda, David Gomez-Almaguer, Rocio Ortiz-Lopez

**Affiliations:** ^1^Departamento de Bioquimica y Medicina Molecular, Facultad de Medicina, Universidad Autonoma de Nuevo Leon, Avenida F. I. Madero, S/N, Col. Mitras Centro, 64460 Monterrey, NL, Mexico; ^2^Molecular Medicine Laboratory, Unidad Academica de Medicina Humana y Ciencias de la Salud, Universidad Autonoma de Zacatecas, Carretera Zacatecas-Guadalajara Km 6, 98160 Ejido la Escondida, ZAC, Mexico; ^3^Servicio de Hematologia, Hospital Universitario “Jose Eleuterio Gonzalez”, Universidad Autonoma de Nuevo Leon, Avenida F. I. Madero, S/N, Col. Mitras Centro, 64460 Monterrey, NL, Mexico; ^4^Instituto Nacional de Medicina Genomica (INMEGEN), Periferico Sur No. 4809, Col. Arenal Tepepan, Delegacion Tlalpan, 14610 Mexico, DF, Mexico; ^5^Mesoamerican Center of Public Health Studies and Disasters (CEMESAD), Universidad Autonoma de Chiapas (UNACH), Avenida Pista Principal esq Pista Secundaria, S/N, 30798 Tapachula, CHIS, Mexico; ^6^Centro de Investigacion y Desarrollo en Ciencias de la Salud, Universidad Autonoma de Nuevo Leon, Carlos Canseco, S/N, Col. Mitras Centro, 64460 Monterrey, NL, Mexico

## Abstract

*Background.* Acute lymphoblastic leukemia type B (B-ALL) is a neoplastic disorder with high mortality rates. The aim of this study was to validate the expression profile of 45 genes associated with signaling pathways involved in leukemia and to evaluate their association with the prognosis of B-ALL. *Methods.* 219 samples of peripheral blood mononuclear cells obtained from 73 B-ALL patients were studied at diagnosis, four, and eight weeks after starting treatment. Gene expression was analyzed by quantitative real-time polymerase chain reaction. *Results.* Normalized delta Cq values of 23 genes showed differences between B-ALL and controls at diagnosis time (*P* values < 0.05). There were significant associations between B-ALL patients relapse/death and the expression levels of IL2RA, SORT1, DEFA1, and FLT3 genes at least in one of the times evaluated (*P* values < 0.05 and odds ratio ranges: 3.73–27). The association between FLT3 deregulation and relapse/death was a constant in the times studied and their overexpression significantly increased the odds of relapse/death in a range of 3.73 and 6.05 among study population (*P* values < 0.05). *Conclusions.* Overexpression of FLT3 and DEFA1 genes retained independent prognostic significance for B-ALL outcome, reflected as increased risks of relapse/death among the study population.

## 1. Background

Acute lymphoblastic leukemia (ALL) is a neoplastic disorder of lymphoid progenitor cells characterized by diverse cytogenetic and molecular abnormalities with peaks of prevalence for 2–5-year-old patients and those older than 50 [1]. Treatment strategies using risk-adapted chemotherapy cure more than 80% of childhood cases, but around 20 to 30% relapse developing serious complications including death [2, 3]. The ALL cases originated from B lymphocyte progenitors represent 85% of childhood and 75% of adult cases, respectively [2, 4]. In Mexico, leukemia mortality represents the second leading cause of death in childhood and adolescence and the 18th in patients during the productive age (15 to 64 years old) [5, 6].

Common cytogenetic subtypes abnormalities such as TEL-AML1 (ETV6-RUNX1)/t(12;21), BCR-ABL (BCR-ABL1)/t(9;22), MLL rearrangements/t(11q23), E2A-PBX1 (TCF3-PBX1)/t(1;19), MYC-IGH/[t(8;14), t(2;8) or t(8;22)], and hyperdiploidy/(50 chromosomes) are typically associated with ALL prognosis and together with age, white blood cell (WBC) count, leukemic cell immunophenotype, and time to respond to therapy define the risk stratification group [7]. However, major prognostic abnormalities in chromosome number or structure are absent in approximately 10–30% of ALL patients, and in a significant number of cases the prediction is not successful [8–10]. Deregulated gene expression of several key cellular pathways has been suggested as a useful tool to refine prognosis and identify novel therapeutic targets in ALL [11]. Though there are many studies of gene expression based on microarray technologies that suggest candidate genes for the prognosis or outcome for ALL patients [11–15], to date all the validation studies have focused on a small number of genes or in strategies tailored to specific disease subgroups.

Attending the need to improve relapse prediction, the aim of this study was the longitudinal validation of a comprehensive gene expression profile of 45 candidate genes involved in key cell signaling pathways and to determine whether their expression was associated with relapse in a group of Mexican B-ALL patients. In this panel, genes and pathways previously reported as altered by expression or copy number variations (CNV), known targets of sequence mutations in ALL, genes commonly mutated in other cancers, and ALL nonrelated genes involved in key signaling pathways were studied [16–18].

## 2. Methods

### 2.1. Patients and Samples

The patients included in this study were newly diagnosed with ALL by cytological examination, peripheral blood, or bone marrow aspirates analysis, and the B lineage lymphocytic precursor was determined by flow cytometry (see Table 1 for the B-ALL cases characteristics). All patients (adults and children's parents or guardians) provided written informed consent for participation and were treated according to the standard protocols of the Hematology Service of the Hospital Universitario “Dr. José E. González”, School of Medicine of the Universidad Autonoma de Nuevo Leon (UANL), Mexico. Patients received an induction to remission regimen with 3 or 4 drugs, based on previously published protocols [19, 20]. Children with B-ALL were stratified into standard and high risk groups according to the accepted criteria (see Additional Table 1 for treatment details in Supplementary Material available online at http://dx.doi.org/10.1155/2015/828145) [8, 21]. The study followed the criteria of the Declaration of Helsinki and was approved by the Institutional Ethics Committee with the reference number BI05-006.

A total of 73 patients provided peripheral blood at the time of diagnosis and at one and two months after the B-ALL diagnosis. All the samples were collected between September 2005 and February 2008 and were followed for 3 years. A total of 225 samples were used in the study, including 219 leukemic (73 at diagnosis, 73 at first, and 73 at second month, resp.) and 6 healthy peripheral blood controls. A complete blood count (CBC) was made for each patient and a molecular screening of BCR-ABL translocation was assessed by quantitative real-time polymerase chain reaction (qRT-PCR) according to standardized protocols of the Unidad de Diagnóstico Molecular del Departamento de Bioquimica y Medicina Molecular de la Facultad de Medicina de la UANL, Mexico.

### 2.2. RNA Isolation and cDNA Synthesis

Mononuclear cells were purified by Histopaque-1077 (Sigma-Aldrich, St. Louis, MO) and preserved in RNAlater Solution (Life Technologies, Carlsbad, CA) at −20°C. Total RNA was extracted from the mononuclear cells using Qiagen RNeasy Mini Kit (Qiagen, West Sussex, UK) according to the manufacturer's protocol. RNA concentration and its quality were determined by measurement of the optical density at 260 nm and relation 260/280, respectively, using NanoDrop Spectrophotometer (NanoDrop Technologies, Wilmington, DE). Samples with a relation of 260/280 > 1.8 were used in the next steps of the analysis. RNA was stored at −80°C until use. cDNA was synthesized from 1.0 *μ*g of total RNA, in a final volume of 20 *μ*L, using SuperScriptTM III First-Strand Synthesis SuperMix and random hexamers (Invitrogen, Carlsbad, CA) according to the manufacturer's protocol. The final cDNA concentration was measured and the samples were stored at −20°C.

### 2.3. Gene Selection and Primer Design

A total of 45 genes representing key signaling pathways and functional processes were selected for this study (Additional Table 2); 32 of them had previously been related to ALL (a list of references of these studies may be consulted in Additional Table 2). In addition, two reference genes RPL13A and HPRT1 were selected as internal quantitative controls. Gene specific primers for real-time qPCR assay were selected from the Primer Bank database (http://pga.mgh.harvard.edu/primerbank/) and provided by Invitrogen. The primer sequences and product sizes for CCR5, IL10, WNT5, FZD3, CTNNB1, GSK3B, JNK, SOS1, BCL11A, JAK2, STAT5, JUN, BCL2A1, IL2RA, CCND2, RXRA, PDE4D, STAT1, CD10, CREB1, FOS, CYLD, RAPGEF2, SORT1, HK2, S100A8, DOCK10, MYH9, PAPD5, CD2, CD3D, CD8A, PBX1, FAT, NKG2-D, HOXA9, CD44, TNFRSF7, NOTCH1, DEFA1, OPAL1, CASP8AP2, PAX5, FLT3, MYC, RPL13A, and HPRT1 are listed in the Additional Table 2. The efficiency and specificity of each primer set were confirmed with standard curves and melting profile evaluation, and efficiency of amplification relative to reference genes was confirmed by standard curve; all the procedures were carried out following the respective standard guidelines reported previously [22].

### 2.4. Quantitative Real-Time PCR

The qRT-PCR analyses were performed with an ABI 7900 high throughput real-time PCR System (Applied Biosystems, Foster City, CA) in a total volume of 20 *μ*L containing 15 ng of cDNA, 1X SYBR Green PCR Master Mix (Applied Biosystems Inc.) and 300 nM of each primer. All samples were analyzed in duplicate, and melting curve analysis was done to confirm the specificity of amplification and lack of primer dimers. The thermal cycle program consisted of an initial denaturation at 95°C for 10 min, followed by an amplification step for 40 cycles of 15 s at 95°C and 1 min at 60°C. Each experiment included two nontemplate controls to detect any template contamination. The 2^−ΔΔCt^ equation was applied to calculate the relative expression of B-ALL samples [23]. The mean of quantification cycle (Cq) of normal blood samples was used as a calibrator.

### 2.5. Statistical Analysis

Simple comparisons of normalized ΔCq values (square of ΔCq values) between B-ALL cases and healthy controls were determined by Student's *t*-test or Mann-Whitney Rank Sum Test. To evaluate the B-ALL diagnosis capacity of selected genes and/or cutoff values of the gene expression, a receiver operating characteristic curve (ROC) analysis was performed. Differences in the gene expression levels between times were evaluated by repeated measures paired *t* test or Wilcoxon Signed Rank Test. Log-Rank Survival Analysis was performed to evaluate differences between survival curves (constructed using event-free survival (EFS) and relapse-free survival (RFS) ALL cases) considering the cutoff values calculated for the genes differentially expressed between times (ROC analysis). In this evaluation the event was defined by relapse or death and the cases that did not experience the event were classified as EFS. EFS was calculated from the date of diagnosis to the last follow-up or the development of an unfavorable event during the 3 years of monitoring. The odds ratio analysis was carried out for groups with positive differences between survival curves; the case grouping was based on the gene expression level for the gene of interest above or equal/below their cutoff calculated previously. The evaluation of the usefulness of IL2RA, SORT1, FLT3, and DEFA1 expression as independent predictors of B-ALL outcome was performed by multivariate logistic regression using EFS/RFS as dependent variable. Finally, a Spearman Rank Order Correlation analysis was used to test the correlation between expression levels of the genes associated with survival and clinical data such as WBC count, percentages of leukemic blasts, and/or diagnosis age. Along the statistical tests, *P* values lower than 0.05 were considered statistically significant. Statistical analysis was performed in the Sigma Plot* v.*11 and GraphPad Prim* v*5.03 software, respectively.

## 3. Results

The study group included 73 B-ALL cases; 75.3% (55) were children (age range: 0 to 18 years) and 24.7% (18) adults (age range: 19 to 44 years). Morphologic, immunologic, and cytogenetic (MIC) working classification grouped 90% of the cases in the three most common B-ALL diagnoses: ALL common, Pre-B-ALL, and B-ALL, respectively (Table 1). 39 cases (53.4%) were classified as high risk at diagnosis and 34 (46.6%) as standard risk. The presence of BCR-ABL rearrangement was found in three cases (4.1%); one of them relapsed during the 3 years of the study. Of the 73 B-ALL patients included 19 (26%) relapsed and 4 (5.5%) died during follow-up.

**Figure 1:**
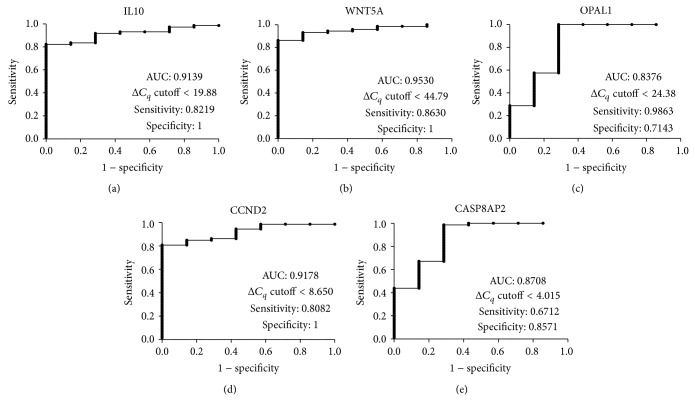
Gene expression classifier for B-ALL diagnosis. ROC curves show the independent accuracy of the 5-probe-set classifier B-ALL diagnosis. (a) ROC curve for IL10, (b) ROC curve for WNT5A, (c) ROC curve for OPAL1, (d) ROC curve for CCND2, and (e) CASP8AP2 ROC curve, respectively.

Additional Table 3 shows the normalized ΔCq values obtained for the study groups. Out of 45 analyzed gene expressions, normalized ΔCq values of 23 genes at diagnosis, 23 at one month, and 28 at second month after starting treatment showed statistical differences between B-ALL cases and healthy controls (*P* values < 0.05). Differences found in 19 genes at diagnosis (IL10, WNT5A, FZD3, JNK, SOS1, JUN, OPAL1, MYC, CCND2, PDE4D, CREB1, FOS, CYLD, RAPGEF2, SORT1, DOCK10, PAPD5, HOXA9, and CASP8AP2) remained constant at least until the third month of follow-up (Additional Table 3). The determination of cutoff values and B-ALL diagnosis capacity for IL10, WNT5A, OPAL1, CCND2, and CASP8AP genes was determined (Figure 1) considering their marked differences with respect to the controls (*P* values ≤ 0.001). The ranges of values obtained for the area under the curve (AUC) and cutoff values for these genes were of 0.8673 to 0.953 and 4.015 to 44.79, respectively. WNT5A has been showed to be the best parameter in the ROC analysis. Considering 44.79 as normalized ΔCq cutoff value for WNT5A, the AUC curve was calculated to be 0.953, with a sensitivity of 0.863, a specificity of 1, positive predictive value (PPV) equal to 1, and a negative predictive value (NPV) of 0.41.

Expression levels for the genes that showed ΔCq statistical differences at least one time between study groups were determined using healthy controls as calibrator (Additional Table 4). Additional Table 4 shows the descriptive statistics of expression level for 30 genes evaluated. In general, ΔCq differences observed between groups for IL10, WNT5A, OPAL1, CCND2, and CASP8AP genes reflected their overexpression with respect to the controls and the median of expression level for each gene was 2.2, 5.4, 7.7, 6.2, and 6.6, for IL10, WNT5A, OPAL1, CCND2, and CASP8AP, respectively.

The results of the identification of genes with statistical differences in their level of expression through time are displayed in Table 2. Considering healthy controls as calibrator, in B-ALL cases the expression levels of 23 genes showed differences between the times evaluated (Table 2). Of these 23 genes, only the differences observed in the expression levels of IL2RA, SORT1, DEFA1, and FLT3 genes in at least one of the studied times were associated with relapse (Table 3). Differences in the median of the FLT3 expression levels between B-ALL cases that presented the event (relapse or ALL-related death) and the event-free group were a constant in the three times studied (*P* < 0.05). When classification of the cohort by diagnosis time or by risk group was considered, FLT3 expression difference between groups was observed only at one month of starting treatment in children (*P* = 0.044) and standard risk (*P* = 0.008) subgroups, respectively. In the same sense, differential expression of IL2RA gene between groups (with/without event) was observed at B-ALL diagnosis time for all the cases and in the children group (*P* values < 0.05). DEFA1 expression presented differences at time 3 for all the cases and in high risk patients (*P* values < 0.05). SORT1 differences were observed only at time 3 in the adults group (*P* = 0.039).

**Figure 2:**
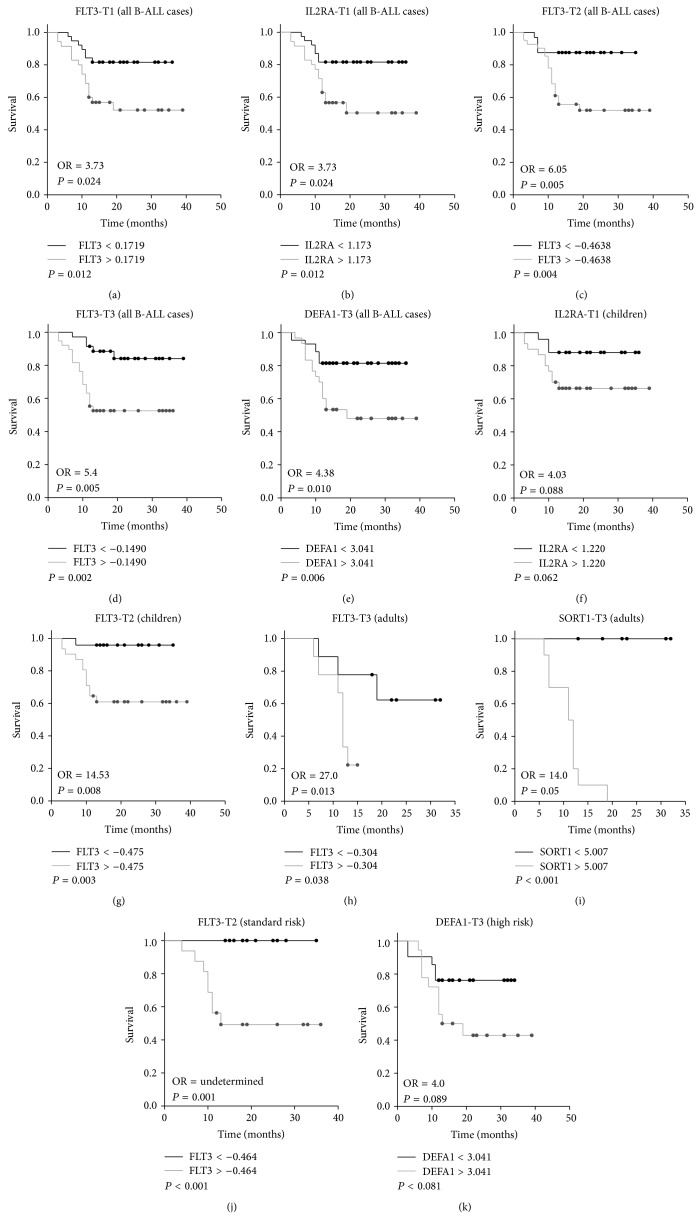
Log-Rank Survival Test. The comparison between survival curves according to specific cutoff values for genes differentially expressed in relapse/death versus event-free cases is showed without classification for (a) FLT3 time 1, (b) IL2RA time 1, (c) FLT3 time 2, (d) FLT3 time 3, and (e) DEFA1 time 3, respectively. Considering B-ALL subgroups, figure shows in children (f) IL2RA time 1 and (g) FLT3 time 2, respectively, and in adults (h) FLT3 time 3 and (i) SORT1 time 3. In mild risk (j) FLT3 time 2 and finally survival curve for (k) DEFA1 time 3 in high risk subgroup, respectively.

To determine the association between expression of IL2RA, SORT1, DEFA1, and FLT3 genes and B-ALL survival, the expression level cutoff values in which the sum of specificity and sensibility were the highest (nearest two) were determined for each gene by ROC analysis. In this test, the B-ALL cases were grouped as event-free patients and those that presented the event (relapse/death). Calculated cutoff values were used to construct and compare survival curves using B-ALL patients whose expression level was above the cutoff and B-ALL cases with expression levels equal to or below the calculated cutoff. The results obtained are shown in Figure 2 and Table 4, respectively. There were statistical differences between survival curves in the study groups (defined by the cutoffs values) for all the genes and times evaluated (*P* values < 0.05); the exception was represented by the IL2RA expression at B-ALL diagnosis time in children and DEFA1 at time 3 in the high risk subgroups, respectively (*P* values > 0.05). The differences in survival times between groups ranged from 6.13 (FLT3-T1) to 22.5 (FLT3-T2-standard risk) months (Table 4). To identify the risk represented by these differences, ORs were calculated preserving the same criteria of classification groups (above or equal to/below cutoff values). B-ALL patients with expression values for IL2RA, SORT1, and FLT3 DEFA1 genes above cutoff values had increased risk of relapse/death ranging from 3.73 to 27 times compared to patients with expression values equal to or below the cutoffs (*P* values < 0.05). FLT3 expression alone was able to detect differences in survival times in a range of 6.13 to 11.8 months and increased relapse/death risk ranging from 3.73 to 6.05 at diagnosis time, at one month, and after two months of starting treatment, respectively. Considering only the B-ALL adults at time 3, an expression level of FLT3-T3 > −0.304 significantly increased the odds of the event relapse/death by 27.0 times (OR = 27; 95% CI: 2.0–368.4, *P* = 0.013), being the highest risk calculated from a specific gene in this study. In this subgroup and at the mentioned cutoff, the observed difference in survival times between groups was 13.43 months (*P* = 0.038). FLT3 expression was also associated with survival in children at time 2 (*P* = 0.003) and in the standard risk group after a month of starting treatment (*P* < 0.001). Independently from FLT3 status, patients with an expression level above 1.173 for IL2RA at B-ALL diagnosis time showed a diminished survival time of 6.35 months (*P* = 0.012) and an increased risk of 3.73 times (95% CI: 1.3–10.72; *P* = 0.024) for relapse/death among the studied population.

After 2 months of starting treatment and compared to B-ALL cases with expression levels below or equal to 3.041, patients with expression level for DEFA1 > 3.041 had increased odds of the event by 4.38 times (95% CI: 1.5–12.5; *P* = 0.01). Finally, in adults at time 3, B-ALL cases with a SORT1 expression level > 5.007 showed differences in their survival curves when compared with cases with expression level lower than or equal to the cutoff for this gene (*P* < 0.001); however, despite an increased risk of relapse/death about 14 times for patients with SORT1 expression level above 5.007, their *P* value reflected only a statistical trend (*P* = 0.05).

To evaluate the usefulness of IL2RA, SORT1, FLT3, and DEFA1 expression as independent predictors of B-ALL outcome a multivariate logistic regression using EFS/RFS as dependent variable was carried out. In the statistical modelling, WBC count, age, gender, age group (children/adult), B-ALL immunophenotype, and SORT-T1, DEFA1-T1, FLT3-T1-3, and IL2RA-T1 expression levels were included as independent variables. The significant B-ALL outcome predictors were WBC count (*P* = 0.023), immunophenotype (OR = 2.6, 95% CI = 1.4–5.1, *P* = 0.004), DEFA1-T1 (OR = 6.1, 95% CI = 1.01–36.9, *P* = 0.049), and FLT3-T2 (OR = 8.6, 95% CI = 1.1–69.3, *P* = 0.043), respectively.

With the aim to test if clinical data including differences in the counts of leukemic blasts cells recovered across samples had an impact on the expression levels of genes associated with survival, a correlation analysis was performed. In this analysis age, B-ALL diagnosis WBC counts, and diagnosis blasts data of 31 participants were included and their correlations with FLT3-T1, SORT-T1, and DEFA1-T1 expression levels were evaluated. Significant correlations between age, WBC, and counts of blasts cells with FLT3-T1, SORT-T1, or DEFA1-T1 expression levels were not observed (*P* values > 0.05). Positive correlation between the following pairs of variables was identified: FLT3-T1/FLT3-T2 (*r* = 0.41, *P* = 0.023), FLT3-T2/FLT3-T3 (*r* = 0.57, *P* = 9.9 × 10^−4^), FLT3-T1/IL2RA-T1 (*r* = 0.69, *P* = 1.1 × 10^−5^), and IL2RA-T1/DEFA-T1 (*r* = 0.72, *P* = 0.008), respectively.

## 4. Discussion

Whilst gene expression profiling studies in ALL have identified gene expression signatures associated with recurrent cytogenetic abnormalities and* in vitro* drug responsiveness, few studies have reported and validated gene expression classifiers of survival [12]. In this study, gene expression biomarkers of relapse-free survival were derived from the gene expression profiles at three different time points on samples of 73 patients with B-ALL (55 children and 18 adults). We evaluated a 45-probe-set containing unique genes related with the principal altered cell signaling pathways in leukemia according to data bases (GeneCards, NCBI, etc.).

Normalized ΔCq values for 19 genes showed consistent differences between cases and controls at least until the second month of follow-up. Interestingly, observed differences were independent of the treatment given. IL10, WNT5A, OPAL1, CCND2, and CASP8AP2 normalized ΔCq presented the most marked differences with respect to the controls; WNT5A was the gene with the best sensitivity/specificity trade-off and therefore the best potential B-ALL diagnosis biomarker evaluated in this study. These findings are relevant because this set of 23 genes may be considered as a B-ALL related signature and their unknown role in the leukemogenesis process should be addressed in future studies. Compared to controls, normalized ΔCq differences of IL10, WNT5A, OPAL1, CCND2, and CASP8AP2 between the study groups were translated in an overexpression of these genes; a high expression of IL10, OPAL1, and CCND2 has been previously reported [24–28]. Contrary to our findings using PBMC samples under expression of CASP8AP2 and WNT5A genes was previously reported in bone marrow specimens from B-ALL cases [29, 30]. This discordance might be related to the different origin of the biological samples evaluated in the studies (PBMC or bone marrow).

Comparisons of gene expression levels through time in B-ALL patients showed consistent differences among a set of 23 genes at least at two of the three time points evaluated and the differences in the expression levels of IL2RA, SORT1, DEFA1, and FLT3 genes in at least one of the times studied were associated with relapse and/or B-ALL-related death. Interestingly, the overexpression of FLT3 at B-ALL diagnosis and at one and two months after starting treatment was associated with relapse/death in our study group; these findings were established without regard to subclassification by clinical characteristics and were significant in children (time 2), adults (time 3), and standard risk (time 2) subgroups. IL2RA overexpression at the time of B-ALL diagnosis was significant in the children's group. In the same sense, while DEFA1 overexpression was significant for all B-ALL cases (time 3) and in the high risk subgroup (time 3), SORT1 overexpression was only different in adults (time 3). Previous results indicate that differences through time in specific gene expression profiles may be useful to identify genes with capacity to pinpoint B-ALL cases with a higher potential risk to relapse.

Grouping the B-ALL cases as event-free patients and those that presented the event (relapse/death), we calculated level expression cutoff values for IL2RA, SORT1, DEFA1, and FLT3 by ROC analysis. These cutoff values allowed us to construct and compare survival curves (and calculate their respective ORs) for groups of B-ALL patients whose expression level was above the calculated cutoff and for patients with gene expression levels equal to or below their respective cutoff. In our study patients with expression values for IL2RA, SORT1, FLT3, and DEFA1 genes above the cutoff values have an increased risk of relapse/death ranging from 3.73 to 27 times compared to B-ALL patients with expression values equal to or below these cutoffs (*P* values < 0.05). The usefulness of FLT3 gene expression to predict an increased risk of relapse/death was more evident than for other genes; since taking into account their cutoffs, this gene was able to identify differences in survival times in a range of 6 to 22 months, and increased relapse/death risks ranging from 3.73 to 27 times. For example, in adults at 2 months after starting treatment the highest risk was observed for patients with an expression level of FLT3-T3 > −0.3036, which presented a decreased mean of survival time of 13.43 months and increased odds of the event (relapse/death) by 27 times (OR = 27; 95% CI: 2.0–368.4, *P* = 0.013). This trend of FLT3 results was valid for B-ALL cases without subclassification at any studied time and after a month of having started the treatment for children and high risk subgroups, respectively. FLT3 gene is a class III receptor tyrosine kinase involved in signaling pathways regulating the proliferation of pluripotent stem cells, early progenitor cells, and immature lymphocytes. Aberrantly expressed FLT3 has been observed at high levels in a spectrum of hematologic malignancies, including 70% to 100% of AML, B-ALL, a fraction of T-Cell ALL, and CML in lymphoid blast crisis [31]; however, this is the first report that evaluated its potential role and association to B-ALL prognosis. These results suggest that the constant monitoring of FLT3 expression profile might lead to a better monitoring in relation to the therapy protocol.

It is important to mention that in this study the presence of BCR-ABL rearrangement was evaluated in the B-ALL cases; only three patients (4.1%) were positive for this molecular abnormality. Considering that the number of patients with this translocation was low and that the screening of other molecular abnormalities was not included in the characterization of the cases, we decided not to remove them from the analysis; however, despite this limitation, the results obtained (without molecular abnormalities classification) showed an important association with B-ALL prognosis, and the expression of three genes demonstrated their usefulness to identify risk of relapse/death of patients with B-ALL (without incorporating additional clinical classification) and, considering that abnormalities in chromosome number or structure are present in 70–90% of the B-ALL cases [8–10], it is possible that the prognosis associations observed may be an independent factor of the presence of molecular rearrangements. However, it is important to note that the role of other no-BCR-ABL abnormalities on the expression signatures of genes evaluated in this protocol represents a significant issue to explore, and therefore its effect on the prognosis value of these genes should be assessed.

We have shown that kinetics of early treatment response evaluated by molecular methods from B-ALL diagnosis at first and/or 2 months after starting the therapy are highly associated with the prognosis of B-ALL patients. Clinical application of these prognosis markers might allow the prospective identification of a significant subgroup of ALL patients with little chance for a cure if treated with contemporary chemotherapeutic regimens, potentially indicating the need for an early stem cell transplant or biologically targeted treatment. Further analysis of these expression profiles coupled with additional comprehensive genomic studies will hopefully lead to the successful identification of novel therapeutic targets and more effective therapies for these patients.

## 5. Conclusions

The kinetics of early treatment response assessed by molecular measures at one and two months after the beginning of therapy is highly associated with leukemia prognosis. Overexpression of FLT3 and DEFA1 genes retained independent prognostic significance for B-ALL outcome, reflected as increased risks of relapse/death among the study population.

## Supplementary Material

Supplementary Material includes the details of the treatment of B-ALL patients involved in the study. This is shown in Additional Table 1. The classification of 45 genes evaluated by cellular signalling pathway and general characteristics of the 47 sets of primers used can be seen in Additional Table 2, while the comparisons of ΔCq values between B-ALL cases and healthy controls in each of the different times studied comes in Additional Table 3. Finally, the descriptive statistics of expression levels for the 30 genes with differences in at least one time between cases and controls may be found in Additional Table 4. All the references for the Supplementary Material, including those related with the previous studies of individual correlations between the 45 genes and cancer were added at the end of the document.

## Figures and Tables

**Table 1:** General characteristics of 73 B-ALL patients.

Characteristic	Number of cases (%)	Mean (range)
Sex		
Male	44 (60.3)	—
Female	29 (39.7)	
Age		
0 to 18	55 (75.3)	7.9 (1–18)
Older than 19	18 (24.7)	28.7 (19–44)
MIC classification		
Early precursor B	2 (2.7)	—
ALL common	22 (30.1)	
ALL pre-B	24 (32.9)	
ALL B	25 (34.3)	
Diagnosis risk		
High	39 (53.4)	—
Regular	34 (46.6)	
White-cell count (/mm^3^)		
<10,000	49 (67.1)	3,676.4 (6.67–9.1 × 10^3^)
10,000–49,999	16 (21.9)	22,025 (1 × 10^4^–4.7 × 10^4^)
50,000–100,000	2 (2.8)	78,755 (7.8 × 10^4^–7.9 × 10^4^)
>100,000	6 (8.2)	212,150 (1 × 10^5^–3.2 × 10^5^)
Percentage of leukemic blasts cells at B-ALL diagnosis	31 (42.5)	75.4 (17.5–95.0)

**Table 2:** Differences in gene expression levels through time in 73 B-ALL patients.

Gene	Times included in the comparison and significant *P* values		
	Time 1/time 2	Time 1/time 3	Time 2/time 3
IL2RA	0.011	ns	ns
IL10	0.012	ns	ns
CD10	0.016	ns	ns
CD3D	0.018	ns	ns
HOXA9	0.041	ns	ns
PAX5	0.006	ns	ns
CTNNB	ns	0.039	ns
GSK3B	ns	0.003	ns
JAK2	ns	0.032	ns
FOS1	ns	0.007	ns
RAPGEF2	ns	0.044	ns
SORT1	ns	0.023	ns
HK2	ns	0.007	ns
PAX5	ns	0.002	ns
DEFA1	ns	0.011	ns
NOTCH1	ns	0.026	ns
S100A8	ns	0.002	ns
	ns	ns	0.037
BCL11A	0.023	ns	ns
	ns	0.012	ns
STAT5	0.017	ns	ns
	ns	0.012	ns
OPAL1	0.04	ns	ns
	ns	0.018	ns
FLT3	<0.001	ns	ns
	ns	0.009	ns
RXRA	0.01	ns	ns
	ns	0.002	ns
BCL2A	0.005	ns	ns
	ns	<0.001	ns
	ns	ns	0.005

ns: nonsignificant, *P* value >0.05.

**Table 3:** Positive associations between gene expression level and relapse/death.

Classification of study population	Gene	Time 1			Time 2			Time 3		
		Event- free group	Event group	*P* value	Event-free group	Event group	*P* value	Event- free group	Event group	*P* value
All the ALL cases	FLT3	−0.049	0.434	0.022^*^	−0.598	−0.075	0.028^*^	−0.242	0.023	0.02^*^
	IL2RA	1.164	1.493	0.038^*^	—	—	—	—	—	—
	DEFA1	—	—	—	—	—	—	1.99	4.012	0.01^*^

Children	FLT3	—	—	—	−0.602	0.025	0.044^*^	—	—	—
	IL2RA	1.193	1.558	0.038^*^	—	—	—	—	—	—

Adults	FLT3	—	—	—	—	—	—	−0.49	0.03	0.046^*^
	SORT1	—	—	—	—	—	—	3.84	5.34	0.039^*^

Standard risk	FLT3	—	—	—	−0.707	−0.025	0.008^*^	—	—	—

High risk	DEFA1	—	—	—	—	—	—	2.33	4.62	0.018^*^

^*^Statistically significant.

**Table 4:** Survival analysis and OR values for differentially expressed genes between B-ALL relapsed patients and event-free cases.

Gene-time (cutoff value)	Group^±^	*N*/events^†^	Survival time (mean, months)	Standard error	95% CI	*P* value^Υ^	OR	95% CI	*P* value^ΥΥ^
FLT3-T1 (0.1719)	1	38/7	31.13	1.803	27.6–34.7	0.012^*^	3.73	1.3–10.72	0.024^*^
	2	35/16	25.003	2.679	19.7–30.3				

IL2RA-T1 (1.173)	1	38/7	31.05	1.829	27.5–34.6	0.012^*^	3.73	1.3–10.72	0.024^*^
	2	35/16	24.71	2.745	19.3–30.1				

FLT3-T2 (−0.4638)	1	32/4	31.47	1.901	27.7–35.2	0.004^*^	6.05	1.8–20.36	0.005^*^
	2	41/19	25.19	2.398	20.5–29.9				

FLT3-T3 (−0.149)	1	35/5	34.83	1.928	31.1–38.6	0.002^*^	5.4	1.7–16.9	0.005^*^
	2	38/18	23.02	2.309	18.5–27.6				

DEFA1-T3 (3.041)	1	43/8	30.84	1.775	27.4–34.3	0.006^*^	4.38	1.5–12.5	0.01^*^
	2	30/15	24.13	2.825	18.6–29.7				

IL2RA-T1-children (1.220)	1	25/3	32.76	2.153	28.5–37.0	0.062	4.03	0.98–16.6	0.088
	2	30/10	28.57	2.857	23.0–34.2				

FLT3-T2-children (−0.475)	1	24/1	33.83	—	—	0.003^*^	14.53	1.7–122.1	0.008^*^
	2	31/12	27.04	2.844	21.5–32.6				

FLT3-T3-adults (−0.304)	1	9/3	24.87	4.128	16.7–33.0	0.038^*^	27	2.0–368.4	0.013^*^
	2	9/7	11.44	1.061	9.4–13.5				

SORT1-T3-Adults (5.007)	1	7/0	—	—	—	<0.001^*^	14	1.1–172.6	0.05
	2	10/10	11	1.193	8.7–13.3				

FLT3-T2-standard risk (−0.464)	1	18/0	—	—	—	<0.001^*^	Undetermined	—	<0.001^*^
	2	16/8	22.51	3.625	15.4–29.6				

DEFA1-T3-high risk (3.041)	1	26/5	27.71	2.780	22.3–33.2	0.081	4.0	1.02–15.72	0.089
	2	28/10	22.79	3.627	15.7–29.9				

^±^Group 1 represents B-ALL cases with a log 2 of expression level ≤ cutoff value (for the indicated gene); in the group 2 those patients with their log 2 of expression level > cutoff for the indicated gene were included. ^Υ^ *P* value for survival curves differences. ^ΥΥ^ *P* value for OR test. ^†^Event indicates relapse or death.

^*^Statistical significance.

## Abbreviations

ALL: Acute lymphoblastic leukemia
CLL: Chronic lymphoblastic leukemia
AML: Acute myeloblastic leukemia
CML: Chronic myeloblastic leukemia
qRT-PCR: Quantitative real-time polymerase chain reaction
Cq: Quantification cycle
PBMC: Peripheral blood mononuclear cells
ROC: Receiver operating characteristic curve
AUC: Area under the curve
EFS: Event-free survival
RFS: Relapse-free survival.
