# Supplementary material for: Identification of Differentially Expressed Genes Associated with Prognosis of B Acute Lymphoblastic Leukemia
Source: Dis Markers. 2015 Feb 24;2015:828145. doi: 10.1155/2015/828145 (PMC4354728; doi:10.1155/2015/828145)
Supplement: Supplementary file 1 — Supplementary Material includes the details of the treatment of B-ALL patients involved in the study. This is shown in Additional Table 1. The classification of 45 genes evaluated by cellular signalling pathway and general characteristics of the 47 sets of primers used can be seen in Additional Table 2, while the comparisons of ΔCq values between B-ALL cases and healthy controls in each of the different times studied comes in Additional Table 3. Finally, the descriptive statistics of expression levels for the 30 genes with differences in at least one time between cases and controls may be found in Additional Table 4. All the references for the Supplementary Material, including those related with the previous studies of individual correlations between the 45 genes and cancer were added at the end of the document. [file 828145.f1.pdf]

Additional Material

Additional Table 1 - Guidelines of B-ALL patient treatment

- Children.

Children with B-ALL were stratified into standard and high-risk groups according to the accepted criteria [8, 19]. Patients received an induction to remission regimen with 3 or 4 drugs, based on previously published protocols [20, 21].Consisting of prednisone 60 mg/m^2, days 1-29, vincristine 1.5 mg/m^2 /week/4 weeks and L-asparaginase 6000 UI/m^2/3 times a week/3 weeks subcutaneously for those in the standard risk group. For children fulfilling the high-risk group criteria, up to four weekly doses of doxorubicin 25 mg/m^2 were additionally administered. In both risk groups, intrathecal chemotherapy was delivered on days 3, 15 and 29. Complete remission was followed by consolidation which in the case of standard risk group included a dose of vincristine, 1.5 mg/m^2, purinethol 75 mg/m^2 p.o. on days 1-28, and intrathecal therapy on days 1, 8 and 15. Children in the high-risk category received 3 additional doses of L asparaginase, cyclophosphamide, 1 gr/m^2 on days 1 and 15, purinethol, 60 mg/m^2 p.o. days 1-28, cytarabine, 75 subcutaneous or i.v. days 1-4, 8-11, 15-18 and 22-25, in addition to intrathecal chemotherapy on days 1,8,15 and 22. Interim maintenance for both groups was the same and included dexamethasone 6 mg/m^2 administered in two doses on days 1-5 and 29-33, vincristine1.5 mg/m^2 days 1 and 29, purinethol 75 mg/m^2 days 1 to 50, methotrexate, 20 mg/m^2 i.v. or i.m. on days 1, 8, 15, 22, 29, 36, 43 and 50, and a single dose of triple intrathecal chemotherapy on day 29. Delayed intensification was administered for both groups in the same way: dexamethasone, 10 mg/m^2 p.o. divided in two doses, days 1-7 and 15-21, vincristine, 1.5 mg/m^2 on days 1,8 and 15, doxorubicin 25 mg/m^2 days 1, 8 and 15 , L-asparaginase 6000UI/m^2/ 3 times a week/2 weeks, cyclophosphamide, 1 gr/m2 i.v. on day 29, cytarabine 75 mg/m^2 i.v. or sc days 29-32 and 36-39, purinethol 60 mg/m^2 on days 29 to 43, and intrathecal chemotherapy delivered on days 1,29 and 36. Maintenance consisted on cycles of 84 days until completing 30 months from initial ALL diagnosis, and included dexamethasone, 6 mg/m^2 divided in two doses on days 1-5, 29-33 and 57-61, vincristine, 1.5 mg/m^2 i.v. on days 1,29 and 57, purinethol 75 mg/m^2 days 1 to 84, weekly methotrexate 20mg/m^2 p.o. beginning on day 1 and intrathecal chemotherapy on days 1 and 29 during the first four cycles, then only on day 1 of the following cycles; oral methotrexate was skipped on the days of intrathecal chemotherapy. Children who failed to achieve a complete remission received two cycles of treatment with a single dose of methotrexate, 1.5 g/m^2 i.v. combined with cytocin arabinoside, 3g/m^2 i.v. each 12 hours per four doses. Children who relapsed during treatment received the same cycles as above, while those relapsing during vigilance and follow-up were given a re-induction to remission with the drugs used for induction, including vincristine, prednisone, doxorubicin and L-asparaginase, as described.

- Adults.

The following table the phases and treatment guidelines used for adults with B-ALL diagnosis included in the protocol are summarized.

Phase	Drug	Dose	Rout	Days
**Induction**	Dexamethasone	24mg/day	IV	1-28
	Vincristine	1.4 mg/m^2	IV	5,12,19,26
	Mitoxantrone	10 mg/m^2	IV	5,6,7
	Intrathecal chemotherapy	Triple	IT	12,19,26
**Consolidation**	Ciclophosphamide			
	L-asparaginase	6000 UI/m^2	IM	1,11,21,31
	Methotrexate	150 mg/m^2	IV	10
	Methotrexate	300 mg/m^2	IV	20
	Intrathecal chemotherapy	Triple	IT	1
**First Reinduction**	Prednisone	60 mg/day	PO	1-15
	Vincristine	1.4 mg/m^2	IV	1,8,15
	Mitoxantrone	10 mg/m^2	IV	1,2,3
	Intrathecal chemotherapy	Triple	IT	1
**Second Reinduction**	Prednisone	60 mg/day	PO	1-15
	Vincristine	1.4 mg/m^2	IV	1,8,15
	Methotrexate	800 mg/m^2	IV	1
	Folinic acid	45 mg/ day	PO	2-6
	Citarabine	2 gr/m^2	IV	15,16
	Intrathecal chemotherapy	Triple	IT	1
**Maintenance during 2 years**	6-mercaptopurine	50 mg/m^2/day	PO	Monday to friday
	Methotrexate	20mg/m^2	PO	Weekly
**Intensification, monthly during first year of maintenance**	Vincristine	1.4 mg/m^2	IV	Monthly
	Prednisone	60 mg/day	PO	Monthly
	Intrathecal chemotherapy	Triple		1

Additional Table 2 – Classification of 45 genes included in the study by cellular signalling pathway and general characteristics of the primers used along the study.

Signaling pathway	Gene	Description	Primer Bank ID	Gene Bank Sequence	Primer Sequence (5'-3')	PCR Product Size (bp)	ALL-related previously*
Cytokine-cytokine receptor interaction/ Hematopoietic cell lineage	CCR5	Chemokine (C-C motif) receptor 5	4502639a1	NM_000579	Fw. AGGGCTGTGAGGCTTATCTTC<br>Rv. CACCTGCATAGCTTGGTCCA	150	Yes [1, 2]
	IL10	Interleukin 10	10835141a1	NM_000572	Fw. ACTTTAAGGGTTACCTGGGTTGC<br>Rv. TCACATGCGCCTTGATGTCTG	111	Yes [3]
	IL2RA	Interleukin 2 receptor, alpha	307047a1	K03122	Fw. GGGGACTGCTCACGTTCATC<br>Rv. GGATCTCTGGCGGGTCATC	72	Yes [4]
	FLT3	fms-related tyrosine kinase 3	1764153a1	U82002	Fw. TCAAGTGCTGTGCATACAATTCC<br>Rv. AGCAGGGTTAAAACGACAATGA	148	Yes [5]
	CD10	CD10 Antigen	4505203a1	NM_000902	Fw. GATCAGCCTCTCGGTCCTTG<br>Rv. TGTTTTGGATCAGTCGAGCAG	134	Yes [6]
	CD2	CD2 Antigen	4502653a1	NM_001767	Fw. TGGACTTGTATCAACACAACCC<br>Rv. GTGTGATGACCCTCTGAGAAAG	112	Yes [6]
	CD3D	CD3d molecule, delta (CD3-TCR complex)	4502669a1	NM_000732	Fw. GCCTGGTACTGGCTACCCT<br>Rv. CGTTCCCTCTACCCATGTGATG	116	No
	CD8A	CD8 Antigen, Alpha Polypeptide (P32)	27886642a1	NM_171827	Fw. ATGGCCTTACCAGTGACCG<br>Rv. AGGTTCCAGGTCCGATCCAG	104	No
	CD44	CD44 Antigen	31191a1	X66733	Fw. CCCTGCTACCAGTACGTCTTC<br>Rv. TGTGGTCAAAAGCCCGTGG	167	Yes [7]
Wnt signaling pathway	WNT5A	Wingless-type MMTV integration site family, member 5A	4507929a1	NM_003392	Fw. ATGGCTGGAAGTGCAATGTCT<br>Rv. ATACCTAGCGACCACCAAGAA	107	Yes [8]
	FZD3	Frizzled family receptor 3	8393378a1	NM_017412	Fw. TTGACTGTGTTCATGGGGCAT<br>Rv. GCTGCTGTCTGTTGGTCATAATG	140	Yes [8]
	CTNNB1	Catenin (cadherin-associated protein),	4503131a1	NM_001904	Fw. GCTACTCAAGCTGATTTGATGGA	120	Yes

		beta 1			Rv. GGTAGTGGCACCAGAATGGATT		[9]
	GSK3B	Glycogen synthase kinase 3 beta	529237a1	L33801	Fw. CGAGGAGAACCCAATGTTTCG<br>Rv. GGTCGGAAGACCTTAGTCCAAG	296	No
MAPK signaling pathway	JUN	Jun proto-oncogene	4758616a1	NM_002228	Fw. TGGAAACGACCTTCTATGACGA<br>Rv. GTTGCTGGACTGGATTATCAGG	242	Yes [10]
	FOS	FBJ murineosteosarcoma viral oncogene homolog	4885241a1	NM_005252	Fw. CGGGCTTCAACGCAGACTA<br>Rv. GGTCCGTGCAGAAGTCCTG	147	Yes [10]
	JNK	C-Jun N-Terminal Kinase 1	4506095a1	NM_002750	Fw. AGCAAGCGTGACAACAATTTTT<br>Rv. GAAATGGTCGGCTTAGCTTCT	175	Yes [11]
	SOS1	Son Of Sevenless Homolog 1	15529996a1	NM_005633	Fw. AAGAGGAAGCGAAGAAACCCT<br>Rv. TCAGCACACATTGCCACTTTAAT	221	No
	RAPGEF2	Rap guanine nucleotide exchange factor (GEF) 2	5911917a1	AL117397	Fw. GTTATGGTGAAAGAACACCGAGA<br>Rv. CACTTCCATTGGGCTAGAAAGAA	183	No
Jak-STAT signaling pathway	JAK2	Janus kinase 2	4826776a1	NM_004972	Fw. GTATCCACCCAACCATGTCTTC<br>Rv. GTTGCTGCCACTGCAATACC	103	Yes [12]
	STAT5	Signal transducer and activator of transcription 5A	21618342a1	NM_003152	Fw. GGTGCAGAGTCCGTGACAG<br>Rv. ACAGGTAGGGACAGAGTCTTC	107	Yes [13]
	STAT1	Signal transducer and activator of transcription 1	6274552a1	NM_007315	Fw. ATGTCTCAGTGGTACGAACTTCA<br>Rv. TGTGCCAGGTACTGTCTGATT	107	Yes [13]
Transcriptional misregulation in cancer	MYC	v-myc myelocytomatosis viral oncogene homolog	312410a1	X00364	Fw. GGGATCGCGCTGAGTATAAAA<br>Rv. GAAGCCCCCTATTCGCTCC	174	Yes [14]
	BCL2A1	BCL2-related protein A1	29691301a1	AY234180	Fw. AACTTCTACGACAGCAAATTGCC<br>Rv. TTGTGCCATTTCCCCCAGC	130	Yes [15]
	RXRA	Retinoid X receptor, alpha	3411009a1	U66306	Fw. CTTCGGTTTTCCGAGCACG<br>Rv. CAGGGACACCAGCCGACTA	149	No
	PBX1	Pre-B-cell leukemia homeobox 1	30582249a1	BT006705	Fw. CATGCTGTTAGCGGAAGGC<br>Rv. CTCCACTGAGTTGTCTGAACC	109	Yes [16]

	HOXA9	Homeobox A9	23097236a1	NM_152739	Fw. TACGTGGACTCGTTCCTGCT<br>Rv. CGTCGCCTTGGACTGGAAG	153	Yes [17]
	PAX5	Paired box 5	9951920a1	NM_016734	Fw. CAGCAGGACAGGACATGGAG<br>Rv. CCTTGATGAGCAAGTTCCACT	108	Yes [18]
Metabolic pathways	PDE4D	Phosphodiesterase 4D, cAMP-specific	32306513a1	NM_006203	Fw. GATTGTGACTCCATTTGCTCAGG<br>Rv. AGGCCATCTCACTGACGGA	251	No
	HK2	Hexokinase 2	15553127a1	NM_000189	Fw. GAGCCACCACTCACCCTACT<br>Rv. ACCCAAAGCACACGGAAGTT	130	No
	HPRT1	Hypoxanthine phosphoribosyl transferase 1	4504483a1	NM_000194	Fw. CCTGGCGTCGTGATTAGTGAT<br>Rv. AGACGTTCAGTCCTGTCCATAA	131	No
	CREB1	cAMP responsive element binding protein 1	33694240a1	AY347527	Fw. CCAGCAGAGTGGAGATGCAG<br>Rv. GGGCTAATGTGGCAATCTGTG	86	Yes [19]
	RPL13A	Ribosomal protein L13a	6912634a1	NM_012423	Fw. CGAGGTTGGCTGGAAGTACC<br>Rv. CTTCTCGGCCTGTTTCCGTAG	121	No
Regulation of actin cytoskeleton/cell-cell communication	MYH9	Myosin, heavy chain 9, non-muscle	30268331a1	AL832639	Fw. GAGCTGGCAAGACGGAGAAC<br>Rv. CTTCCTTGGCTTGGCGGATA	282	No
	FAT	FAT tumor suppressor homolog 1	4885229a1	NM_005245	Fw. GGGAGACATTTGGCTTTGCTC<br>Rv. ATGTAAACACCCATCTTGACAGG	185	Yes [20]
Apoptosis Pathway	BCL11A	B-cell CLL/lymphoma 11A (zinc finger protein)	12150278a1	AF080216	Fw. CTCTCCCGATGTGAACCGAG<br>Rv. TTGCATTGTTTCCGTTTGTGC	263	Yes [21]
	CASP8AP2	Caspase 8 associated protein 2	6912288a1	NM_012115	Fw. TCATAGCACTTCACTGCCAAAT<br>Rv. GAGAACGTGACCAAACACCAT	122	Yes [22]
	OPAL1	Outcome Predictor In Acute Leukemia 1	33563366a1	NM_017787	Fw. ATGCCTTTCCTTTTGGGTCTTAG<br>Rv. CAGGTTGATTTCATGTTGCCG	243	Yes [23]
	TNFRSF7	Tumor Necrosis Factor Receptor Superfamily, Member 7	4507587a1	NM_001242	Fw. CAGAGAGGCACTACTGGGCT<br>Rv. TGCAAGGATCACACTGAGCAG	114	Yes [24]
Cell cycle	CCND2	Cyclin D2	4502617a1	NM_001759	Fw. CTACCTTCCGCAGTGCTCCTA	163	Yes

regulation					Rv. CCCAGCCAAGAAACGGTCC		[25]
	S100A8	S100 calcium binding protein A8	21614544a1	NM_002964	Fw. TGATAAAGGGGAATTTCCATGCC<br>Rv. ACACTCGGTCTCTAGCAATTTCT	65	Yes [26]
	DOCK10	Dedicator of cytokinesis 10	6683721a1	AB014594	Fw. AAAGACCTACCGGAATGATCCT<br>Rv. CACATGCCACTGGGAACTATAAA	184	No
	PAPD5	PAP associated domain containing 5	21739473a1	AL833922	Fw. GTGTTTGGGAAGTGGGAGAAC<br>Rv. CGACTTTGTGTTTCCGAAGAGC	67	No
	CYLD	Cylindromatosis (turban tumor syndrome)	14165258a1	NM_015247	Fw. TCAGGCTTATGGAGCCAAGAA<br>Rv. ACTTCCCTTCGGTACTTTAAGGA	129	Yes [27]
Notch signaling pathway	NOTCH1	Notch Homolog 1, Translocation-Associated	7019819a1	AK000012	Fw. CACACACGGAGGCATCCTAC<br>Rv. GCTGGAGCATCTTCTTCGGAA	119	Yes [27, 28]
Other	NKG2-D	NK Cell Receptor D	6679052a1	NM_007360	Fw. GAGTGATTTTTCAACACGATGGC<br>Rv. ACAGTAACTTTCGGTCAAGGGAA	208	No
	DEFA1	Defensin, alpha 1	4758146a1	NM_004084	Fw. TCCCTTGCATGGGACGAAAG<br>Rv. GGTTCCATAGCGACGTTCTCC	111	Yes [29]
	SORT1	Sortilin 1	17149834a1	NM_002959	Fw. TGATCTCAGAGGCTCAGTATCC<br>Rv. CAGGACCAATAGCCATGCCAA	212	No

* This column indicates previous studies in which an evaluation of the indicated gene in ALL patients has been realized. The complete references list is shown at the bottom of this file.

References listed in the table

1. Durig J, Schmucker U, Duhrsen U: **Differential expression of chemokine receptors in B cell malignancies**. *Leukemia* 2001, **15**(5):752-756.
2. Wong S, Fulcher D: **Chemokine receptor expression in B-cell lymphoproliferative disorders**. *Leuk Lymphoma* 2004, **45**(12):2491-2496.
3. Wu S, Gessner R, Taube T, von Stackelberg A, Henze G, Seeger K: **Expression of interleukin-10 splicing variants is a positive prognostic feature in relapsed childhood acute lymphoblastic leukemia**. *J Clin Oncol* 2005, **23**(13):3038-3042.
4. Nakase K, Kita K, Miwa H, Nishii K, Shikami M, Tanaka I, Tsutani H, Ueda T, Nasu K, Kyo T *et al*: **Clinical and prognostic significance of cytokine receptor expression in adult acute lymphoblastic leukemia: interleukin-2 receptor alpha-chain predicts a poor prognosis**. *Leukemia* 2007, **21**(2):326-332.
5. de Deus DM, de Souza PR, Muniz MT: **High FLT3 expression and IL10 (G1082A) polymorphism in poor overall survival in calla acute lymphoblastic leukemia**. *Mol Biol Rep* 2013, **40**(2):1609-1613.
6. Bayram I, Erbey F, Komur M, Kibar F, Tanyeli A: **Flow cytometry results at diagnosis and relapse in childhood acute lymphoblastic leukemia**. *Asian Pac J Cancer Prev* 2010, **11**(5):1321-1324.
7. Kamazani FM, Bahoush GR, Aghaeipour M, Vaeli S, Amirghofran Z: **CD44 and CD27 expression pattern in B cell precursor acute lymphoblastic leukemia and its clinical significance**. *Med Oncol* 2013, **30**(1):359.
8. Khan NI, Bradstock KF, Bendall LJ: **Activation of Wnt/beta-catenin pathway mediates growth and survival in B-cell progenitor acute lymphoblastic leukaemia**. *Br J Haematol* 2007, **138**(3):338-348.
9. Nygren MK, Dosen G, Hystad ME, Stubberud H, Funderud S, Rian E: **Wnt3A activates canonical Wnt signalling in acute lymphoblastic leukaemia (ALL) cells and inhibits the proliferation of B-ALL cell lines**. *Br J Haematol* 2007, **136**(3):400-413.
10. Volm M, Sauerbrey A, Stammler G, Zintl F: **Detection of fos, jun and ras in newly-diagnosed childhood acute lymphoblastic-leukemia by immunocytochemistry and PCR**. *Int J Oncol* 1994, **4**(6):1251-1256.
11. Leung KT, Li KK, Sun SS, Chan PK, Ooi VE, Chiu LC: **Activation of the JNK pathway promotes phosphorylation and degradation of BimEL--a novel mechanism of chemoresistance in T-cell acute lymphoblastic leukemia**. *Carcinogenesis* 2008, **29**(3):544-551.
12. Roll JD, Reuther GW: **CRLF2 and JAK2 in B-progenitor acute lymphoblastic leukemia: a novel association in oncogenesis**. *Cancer Res* 2010, **70**(19):7347-7352.
13. Gouilleux-Gruart V, Gouilleux F, Desaint C, Claisse JF, Capiod JC, Delobel J, Weber-Nordt R, Dusanter-Fourt I, Dreyfus F, Groner B *et al*: **STAT-related transcription factors are constitutively activated in peripheral blood cells from acute leukemia patients**. *Blood* 1996, **87**(5):1692-1697.
14. Delgado MD, Leon J: **Myc roles in hematopoiesis and leukemia**. *Genes Cancer* 2010, **1**(6):605-616.
15. Nagy B, Lundan T, Larramendy ML, Aalto Y, Zhu Y, Niini T, Edgren H, Ferrer A, Vilpo J, Elonen E *et al*: **Abnormal expression of apoptosis-related genes in haematological malignancies: overexpression of MYC is poor prognostic sign in mantle cell lymphoma**. *Br J Haematol* 2003, **120**(3):434-441.
16. Zhang R, Liao J, Li G, Sun HQ, Shi YJ, Yang JY: **[Real-time quantitative detection of E2A-PBX1 fusion gene in children with acute lymphoblastic leukemia and its clinical application in minimal residual disease monitoring]**. *Zhongguo Dang Dai Er Ke Za Zhi* 2013, **15**(6):440-443.
17. Whelan JT, Ludwig DL, Bertrand FE: **HoxA9 induces insulin-like growth factor-1 receptor expression in B-lineage acute lymphoblastic leukemia**. *Leukemia* 2008, **22**(6):1161-1169.
18. Shang Z, Zhao Y, Zhou K, Xu Y, Huang W: **PAX5 alteration-associated gene-expression signatures in B-cell acute lymphoblastic leukemia**. *Int J Hematol* 2013, **97**(5):599-603.
19. Wen C, Ma FT, Wan WQ: **[Expression of CREB/Bcl-2 in bone marrow mononuclear cells of children with acute leukemia]**. *Zhongguo Dang Dai Er Ke Za Zhi* 2010, **12**(3):177-180.
20. de Bock CE, Ardjmand A, Molloy TJ, Bone SM, Johnstone D, Campbell DM, Shipman KL, Yeadon TM, Holst J, Spanevello MD *et al*: **The Fat1 cadherin is overexpressed and an independent prognostic factor for survival in paired diagnosis-relapse samples of precursor B-cell acute lymphoblastic leukemia**. *Leukemia* 2012, **26**(5):918-926.
21. Agueli C, Cammarata G, Salemi D, Dagnino L, Nicoletti R, La Rosa M, Messana F, Marfia A, Bica MG, Coniglio ML *et al*: **14q32/miRNA clusters loss of heterozygosity in acute lymphoblastic leukemia is associated with up-regulation of BCL11a**. *Am J Hematol* 2010, **85**(8):575-578.
22. Flotho C, Coustan-Smith E, Pei D, Iwamoto S, Song G, Cheng C, Pui CH, Downing JR, Campana D: **Genes contributing to minimal residual disease in childhood acute lymphoblastic leukemia: prognostic significance of CASP8AP2**. *Blood* 2006, **108**(3):1050-1057.
23. Holleman A, den Boer ML, Cheok MH, Kazemier KM, Pei D, Downing JR, Janka-Schaub GE, Gobel U, Graubner UB, Pui CH *et al*: **Expression of the outcome predictor in acute leukemia 1 (OPAL1) gene is not an independent prognostic factor in patients treated according to COALL or St Jude protocols**. *Blood* 2006, **108**(6):1984-1990.
24. Gandemer V, Rio AG, de Tayrac M, Sibut V, Mottier S, Ly Sunnaram B, Henry C, Monnier A, Berthou C, Le Gall E *et al*: **Five distinct biological processes and 14 differentially expressed genes characterize TEL/AML1-positive leukemia**. *BMC Genomics* 2007, **8**:385.
25. Clappier E, Cuccuini W, Cayuela JM, Vecchione D, Baruchel A, Dombret H, Sigaux F, Soulier J: **Cyclin D2 dysregulation by chromosomal translocations to TCR loci in T-cell acute lymphoblastic leukemias**. *Leukemia* 2006, **20**(1):82-86.
26. Spijkers-Hagelstein JA, Schneider P, Hulleman E, de Boer J, Williams O, Pieters R, Stam RW: **Elevated S100A8/S100A9 expression causes glucocorticoid resistance in MLL-rearranged infant acute lymphoblastic leukemia**. *Leukemia* 2012, **26**(6):1255-1265.
27. D'Altri T, Gonzalez J, Aifantis I, Espinosa L, Bigas A: **Hes1 expression and CYLD repression are essential events downstream of Notch1 in T-cell leukemia**. *Cell Cycle* 2011, **10**(7):1031-1036.
28. Tosello V, Ferrando AA: **The NOTCH signaling pathway: role in the pathogenesis of T-cell acute lymphoblastic leukemia and implication for therapy**. *Ther Adv Hematol* 2013, **4**(3):199-210.
29. Te Kronnie G, Bicciato S, Franceschini L, Accordi B, Delliorto MC, Rinaldi A, Pession A, Barisone E, Conter V, Locatelli F *et al*: **Validation by RQ-PCR and flow cytometry of alpha-defensin1-3 (DEFA1-3) overexpression in relapsed and refractory acute lymphoblastic leukemia**. *Oncol Rep* 2006, **15**(2):341-346**.**

Additional Table 3 –Comparisons of ΔCt values between B-ALL cases and healthy controls.

Gene	Controls normalized ΔCt	ALL cases: Time 1		ALL cases: Time 2		ALL cases: Time 3	
		Normalized ΔCt	P value	Normalized ΔCt	P value	Normalized ΔCt	*P* value
CCR5	2.90	4.60	0.16	4.50	0.005*	4.43	0.01*
IL10	35.60	12.75	< 0.001*	13.23	0.003*	11.61	0.002*
WNT5A	84.24	20.22	< 0.001*	21.10	< 0.001*	17.10	< 0.001*
FZD3	55.20	14.90	0.002*	15.98	0.008*	17.20	0.003*
CTNNB1	5.86	1.12	0.054	0.86	0.029*	0.76	0.016*
GSK3B	30.51	26.55	0.67	23.91	0.268	21.62	0.227
JNK	40.68	15.18	0.022*	14.10	0.01*	14.34	0.012*
SOS1	14.26	4.85	0.012*	5.60	0.006*	4.20	0.003*
BCL11A	54.58	21.51	0.039*	31.61	0.077	30.90	0.134
JAK2	3.72	1.92	0.212	0.79	0.039*	1.00	0.029*
STAT5	7.81	1.82	0.068	0.56	0.011*	0.73	0.004*
JUN	23.18	6.17	0.008*	6.17	0.011*	6.00	0.009*
BCL2A1	0.05	1.08	0.017*	0.85	0.163	1.00	0.134
OPAL1	133.76	5.51	0.003*	3.10	0.001*	4.00	0.001*
FLT3	3.80	4.61	1	6.20	0.268	5.32	0.299
MYC	3.43	1.39	0.007*	1.20	0.011*	1.30	0.01*
IL2RA	14.50	6.29	0.173	7.00	0.369	7.00	0.307
CCND2	46.71	1.16	< 0.001*	1.79	< 0.001*	1.51	< 0.001*
RXRA	10.81	2.51	0.433	1.43	0.085	1.43	0.032*
PDE4D	111.20	44.20	0.032*	44.00	0.033*	43.20	0.025*
STAT1	7.81	1.65	0.196	1.12	0.059	0.77	0.032*
CD10	22.70	6.48	0.085	7.00	0.19	6.00	0.134
CREB1	39.70	1.02	0.009*	0.58	0.005*	0.98	0.006*
FOS	22.34	4.82	0.019*	4.68	0.011*	4.10	0.013*
CYLD	7.81	0.90	0.007*	0.83	0.014*	0.44	0.002*
RAPGEF2	112.83	7.66	0.004*	7.10	0.004*	5.40	0.002*
SORT1	35.62	7.00	0.013*	7.00	0.008*	4.32	0.003*
HK2	41.00	27.11	0.134	21.00	0.085	15.84	0.031*
S100A8	7.33	5.40	0.291	6.30	0.464	8.90	0.798
DOCK10	17.30	1.11	0.045*	0.96	0.031*	0.98	0.015*
MYH9	5.10	5.30	0.721	4.88	0.683	4.52	0.454
PAPD5	4.85	2.10	0.028*	1.80	0.031*	1.59	0.02*
CD2	8.91	0.93	0.029*	1.01	0.054	0.50	0.014*
CD3D	24.90	2.65	0.24	2.50	0.153	1.73	0.046*
CD8A	24.31	26.50	0.812	24.12	0.609	20.48	0.323
PBX1	15.64	21.71	0.507	25.20	0.634	18.33	0.695
FAT	26.60	9.50	0.025*	11.41	0.168	9.41	0.074

NKG2-D	15.10	2.43	0.404	1.54	0.158	1.56	0.057
HOXA9	6.24	11.72	0.046*	12.90	0.032*	10.72	0.026*
CD44	55.01	51.20	0.444	49.42	0.358	43.33	0.063
TNFRSF7	0.57	0.53	0.485	1.10	0.838	0.68	0.772
PAX5	9.30	4.60	0.099	5.10	0.385	5.91	0.376
DEFA1	8.10	5.06	0.276	6.10	0.276	5.60	0.291
CASP8AP2	133.60	2.94	0.001*	3.34	0.003*	2.83	0.002*
NOTCH1	1.10	2.84	0.202	2.40	0.307	0.33	0.276

P values obtained from the comparison between ALL cases and controls by time.

ΔCt normalized values corresponds to square of ΔCt values (see methods section for details).

*Statistical significance

Additional Table 4 – Descriptive statistics of gene expression levels for the 30 genes with differences in at least one time between cases and controls.

Gene	Time 1			Time 2			Time 3		
	Range max	Range min	Median	Range max	Range min	Median	Range max	Range min	Median
BCL11A	34.75	-3.11	3.70	0.26	-3.21	-0.67	0.14	-3.64	-0.49
CASP8AP2	9.26	2.0	6.70	8.31	-0.32	6.59	8.08	0.86	6.73
CCND2	29.53	-2.63	6.49	34.62	-1.74	6.43	33.92	-1.44	6.08
CREB1	6.32	-11.92	4.58	32.90	-3.28	4.83	7.10	-4.86	4.61
CYLD	3.99	-2.64	2.26	28.67	-5.35	2.32	3.90	-2.32	2.47
DOCK10	33.13	-5.22	3.98	5.90	-7.64	3.82	31.39	-3.81	3.91
FOS	18.08	-10.14	-3.64	1.36	-9.50	-3.42	22.47	-11.77	-2.69
FZD3	6.65	-3.28	1.99	6.63	-3.63	1.85	4.06	-4.10	2.22
HOXA9	1.59	-6.67	-0.76	1.46	-8.08	-0.93	5.01	-8.08	-0.61
IL10	7.05	-3.28	2.25	3.93	-2.95	2.14	4.12	-2.09	2.44
JNK	34.53	-4.30	2.32	33.5	-4.69	2.46	32.79	-2.67	2.40
JUN	4.65	-1.14	2.54	4.88	-1.15	2.54	4.15	-3.16	2.59
MYC	27.90	-1.65	1.08	25.25	-0.75	1	5.03	-0.70	0.95
OPAL1	9.27	4.66	7.47	9.66	5.38	8.07	9.35	5.45	7.80
PAPD5	2.64	-5.19	0.56	1.85	-6.78	0.66	25.68	-11.88	0.76
PDE4D	37.63	-1.17	2.88	36.6	-2.36	2.90	35.89	-2.73	2.99
RAPGEF2	8.33	-2.1	5.81	9.29	-1.51	5.92	31.04	0.3	6.25
SOS1	29.99	-12.68	1.76	6.42	-7.77	1.58	30.43	-4.61	1.83
WNT5A	8.50	-2.41	5.39	7.69	-2.51	5.30	7.95	-1.89	5.75
CD2	4.90	-1.42	2.59	NS	NS	NS	4.74	-10.34	2.85
BCL2A1	1.63	-9.25	-0.66	NS	NS	NS	NS	NS	NS
FAT	4.80	-3.95	1.98	NS	NS	NS	NS	NS	NS
HK2	NS	NS	NS	NS	NS	NS	29.28	-5.80	2.83
CD3D	NS	NS	NS	NS	NS	NS	30.25	-3.55	2.33
RXRA	NS	NS	NS	NS	NS	NS	4.13	-6.79	1.28
STAT1	NS	NS	NS	NS	NS	NS	5.71	-7.85	2.69
CTNNB1	NS	NS	NS	2.52	-0.97	1.03	2.47	-3.50	1.08
JAK2	NS	NS	NS	3.03	-7.00	1.23	3.42	-9.68	1.15
SORT1	NS	NS	NS	33.42	-4.95	3.53	29.79	-5.68	4.10
STAT5	NS	NS	NS	5.303	-7.39	1.66	28.85	-5.33	1.48

All the values shown in the table represent the Log 2 of gene expression level.
NS: Non significant ($P > 0.05$)
